# Multilingual publishing in the social sciences and humanities: A seven‐country European study

**DOI:** 10.1002/asi.24336

**Published:** 2020-01-22

**Authors:** Emanuel Kulczycki, Raf Guns, Janne Pölönen, Tim C. E. Engels, Ewa A. Rozkosz, Alesia A. Zuccala, Kasper Bruun, Olli Eskola, Andreja Istenič Starčič, Michal Petr, Gunnar Sivertsen

**Affiliations:** ^1^ Scholarly Communication Research Group Adam Mickiewicz University in Poznań Poznań Poland; ^2^ Centre for R&D Monitoring, Faculty of Social Sciences University of Antwerp Antwerp Belgium; ^3^ Federation of Finnish Learned Societies Helsinki Finland; ^4^ Department of Communication University of Copenhagen Copenhagen Denmark; ^5^ Ministry of Higher Education and Science, Agency for Science and Higher Education Copenhagen Denmark; ^6^ CSC – IT Center for Science Espoo Finland; ^7^ Faculty of Civil and Geodetic Engineering University of Ljubljana Ljubljana Slovenia; ^8^ Faculty of Education University of Primorska Koper Slovenia; ^9^ Institute of Psychology and Education Kazan Federal University Kazan Russia; ^10^ Research Office, Masaryk University Brno Czech Republic; ^11^ Nordic Institute for Studies in Innovation, Research and Education Oslo Norway

## Abstract

We investigate the state of multilingualism across the social sciences and humanities (SSH) using a comprehensive data set of research outputs from seven European countries (Czech Republic, Denmark, Finland, Flanders [Belgium], Norway, Poland, and Slovenia). Although English tends to be the dominant language of science, SSH researchers often produce culturally and societally relevant work in their local languages. We collected and analyzed a set of 164,218 peer‐reviewed journal articles (produced by 51,063 researchers from 2013 to 2015) and found that multilingualism is prevalent despite geographical location and field. Among the researchers who published at least three journal articles during this time period, over one‐third from the various countries had written their work in at least two languages. The highest share of researchers who published in only one language were from Flanders (80.9%), whereas the lowest shares were from Slovenia (57.2%) and Poland (59.3%). Our findings show that multilingual publishing is an ongoing practice in many SSH research fields regardless of geographical location, political situation, and/or historical heritage. Here we argue that research is international, but multilingual publishing keeps locally relevant research alive with the added potential for creating impact.

## 1 INTRODUCTION

Researchers from the social sciences and humanities (SSH) who study culture and society often publish in local languages. Language plays a key role in terms of influencing debates and decision‐making related to issues of cultural heritage, migration, and/or public administration (Sivertsen, 2018). A complete picture of the SSH publishing landscape therefore requires coverage of journals in all languages, not just those typically covered by English‐biased databases, like Scopus and Web of Science (WoS; Mongeon & Paul‐Hus, 2016). Often this level of multilingual coverage can only be found in national bibliographic databases (Sīle et al., 2018). However, the dominant language of academia is English, and there are two effects: first, that it greatly facilitates international communication and the exchange of research results (Gordin, 2015), and second, it can prohibit researchers from non‐Anglophone countries from making significant contributions to top‐tier publication channels (Ammon, 2012; Hyland, 2016).

In the last 20 years various countries worldwide—for example, Australia, the Czech Republic, Finland, Norway, Poland, and the UK—have chosen to implement performance‐based research funding systems (Aagaard, 2015; Hicks, 2012; Kulczycki, 2017) and incentive regimes (Franzoni, Scellato, & Stephan, 2015; Quan, Chen, & Shu, 2017) linked to the publication behavior of researchers (Neff, 2018; Rochmyaningsih, 2019). In many countries (Ochsner, Kulczycki, & Gedutis, 2018; Sīle et al., 2018) research articles published in English represent a standard of research quality and internationalization. Yet, when information sources, like the WoS and Scopus, are used for research evaluation, local language publications produced in the SSH tend to be neglected (Liu, 2017). Neglected or undervalued research is less likely, then, to fulfill its responsibilities toward society, or create localized impacts.

Sivertsen (2018) argues that local language use in scholarship is needed to foster engagement with stakeholders and the public. However, if evaluation regimes influence publication practices and modify research agendas (Bianco, Gras, & Sutz, 2016), researchers may choose to move away from locally relevant research toward decontextualized approaches of interest to English‐language audiences (López Piñeiro & Hicks, 2015). In fact, different languages and communication channels have an impact on different audiences (Hicks, 2005). As Chavarro, Tang, and Ràfols (2017) show, non‐English journals serve communication functions that are different than mainstream English ones: they give researchers opportunities for initiation into publication, and publish topics that are not well covered by mainstream channels. Moreover, language plays a role in shaping how we tend to think about abstract concepts (Boroditsky, 2001). Thus, publishing in more than one language not only reaches a wider audience but supports a diverse perspective on research.

The purpose of this article is to make use of the most comprehensive data set of SSH researchers and their peer‐reviewed journal articles thus far, in order to investigate the current state of multilingualism in seven European countries. We provide evidence that for SSH researchers multilingualism is often vital, regardless of geographical location and field.

Diverse initiatives have been established to improve research evaluation. The San Francisco Declaration on Research Assessment (https://sfdora.org) highlights that the scientific content of publications is more important than the publication metrics of the journals in which they were published. In the Metric Tide report, Wilsdon et al. (2015) argue that evaluation should support the diversity and plurality of research. In the Leiden Manifesto, Hicks, Wouters, Waltman, de Rijcke, and Rafols (2015) emphasize that excellence in locally relevant research should be protected. Yet all these recommendations overlook one key factor in the communication of locally relevant research results: publication language. The Helsinki Initiative on Multilingualism in Scholarly Communication (http://helsinki-initative.org) has now also been established (April 3, 2019) to support the dissemination of research results in all languages.

A balanced approach to multilingualism (Sivertsen, 2018) benefits society, when it supports the use of language in a holistic manner without setting priorities in scholarly communication. Hence, the number of languages in which researchers can communicate their results is essential. Previous studies have yet to focus more critically on the role of publication language on research impact (Engels, Istenič Starčič, Kulczycki, Pölönen, & Sivertsen, 2018; Engels, Ossenblok, & Spruyt, 2012; Kulczycki et al., 2018; Mañana‐Rodríguez & Giménez‐Toledo, 2011), with the exception of two, published by Verleysen and Weeren (2016a, 2016b). This is because research is often implemented at the publication level rather than at the level of the researcher, where language resides. National databases, designed to cover all topics and languages comprehensively, are therefore essential for promoting a balanced approach to multilingualism in scholarly communication.

The purpose of this study is to assess language patterns in journal articles published by researchers from seven European countries across a variety of SSH fields. The data set included 164,218 journal articles from the years 2013–2015, produced by 51,063 researchers from the Czech Republic, Denmark, Finland, Flanders (Belgium), Norway, Poland, and Slovenia. Utilizing this data set, we investigate the number of languages in which the researchers have communicated their research.

Earlier (Kulczycki et al., 2018), we have shown that it is possible to achieve a fuller picture of scholarly communication utilizing national databases built upon institutional research information systems. The data used in our earlier study included peer‐reviewed journal articles registered in the comprehensive databases of seven countries: the National Registry of RD & I Outputs (RIV) for the Czech Republic, the Danish Bibliometric Research Indicator (BFI) for Denmark, the Flemish Academic Bibliographic Database for the Social Sciences and Humanities (VABB‐SHW) for Flanders (Belgium), the VIRTA Publication Information Service for Finland, the Norwegian Science Index (NSI) for Norway, the Polish Scholarly Bibliography (PBN) for Poland, the Slovenian Current Research Information System (SICRIS) for Slovenia. Sīle et al. (2018) describe their framework, structure, and coverage, and for each national database we have recently identified the local or “arterial” language(s) of a region or a country. For instance, Czech in the Czech Republic, and Danish in Denmark (see the Methods section).

## 2 METHODS

### 2.1 *Data set*

Our current data set now includes a list of unique SSH researchers, a list of unique peer‐reviewed journal articles, and a list of researchers paired with article IDs, linking all researchers to the articles that each has authored. The list of articles is unique per country, in that one article is represented by one bibliographical record even if it is coauthored by more than one researcher in that country.

### 2.2 *Researchers*

The set of researchers included in this study represents all SSH researchers affiliated with our country‐specific universities or research institutions. In some cases, the data recorded by an institution in a national database differs. For instance, in some countries publications of PhD researchers are not registered (see Table S1).

### 2.3 *Field classification*

All researchers were further classified according to their field (see more about the process in the Supplementary Information and in Table S2). Thus, each article receives a count on the basis of the researcher's assigned field. All national field classifications used here have also been mapped to the Organization for Economic Co‐Operation and Development Revised Fields of Science and Technology classification (OECD FOS classification; OECD, 2007).

### 2.4 *Peer‐reviewed articles*

Since each national database applies a different method for identifying peer‐reviewed articles, we have chosen to follow two (see Table S3). The first one relies on authors' (or universities') self‐reports concerning peer‐reviewed articles (for example, Finland). The other method identifies peer‐reviewed articles on the basis of authority lists (for example, the Bibliometric Research Indicator [BFI] in Denmark).

### 2.5 *Regression model*

We constructed a binary logistic regression model (logit model) to examine the predictors of multilingualism. A chi‐square statistic was used in order to investigate the extent to which the model predicts the dependent variable (Field, Miles, & Field, 2012).

### 2.6 *Publication counting methods*

A whole‐counting method was used for the articles from each of the databases. Our analyses were conducted on three different levels, each of which may influence the results. At the national level, (a) each article is counted once and (b) all articles coauthored by authors affiliated with two or more of our seven countries are counted for each country. At the field level for a country, every article is counted once for a given field. Given that authors have been assigned to fields, rather than individual articles, some articles are counted more than once for each country. In other words, if an article is coauthored by two authors from two different fields, then this article is counted once separately for each field. At the researcher level for a country, every article is counted once on the basis of authorship. For example, if an article is coauthored by four researchers from a given country (two from the humanities and two from the social sciences) then we count as follows: one article at the national level, two articles at the field level (one in the humanities and one in the social sciences), and one article per researcher at the research level (four articles if we sum the total number of researchers).

### 2.7 *Languages*

Language information for each article is contained in each national database. In cases where this information is missing, the language was determined manually or automatically using the publication title. If more than one language is assigned to an article, we chose to use the first one. In this study we classify publication language as follows: English, local language, and other. The local languages are Czech (RIV, Czech Republic), Danish (BFI, Denmark), Dutch (VABB‐SHW, Flanders), Finnish and Swedish (VIRTA, Finland), Norwegian (NSI, Norway), Polish (PBN, Poland), and Slovene (Slovenian Current Research Information System, Slovenia). Note that we refer to Flanders as a country in the article, even though it is technically a region in the country of Belgium with a high degree of autonomy.

### 2.8 *Limitations of the study*

One of the main limitations to this study concerns the way in which each national database has been designed, and how the data collected influences reported percentages. This means that all exact numbers and shares that are reported may be biased as a result of how comprehensive the database is, or how its records are created based on underlying definitions.

Nevertheless, all of our national databases have been designed to cover each country's peer‐reviewed publications (Sīle et al., 2018); therefore, publication patterns and trends can be assessed comparatively. In our section of Supplementary Information, we provide details about how researchers are defined, which field classification is used, and what methods of identifying peer‐reviewed articles are implemented by each country. On the one hand, database differences are inevitable, where some are more than just bibliographic resources and may be used to serve other purposes (for example, as a data source for identifying where to distribute state‐funding). On the other hand, our use of national databases for this study clearly shows how we can observe actual publication practices beyond the scope of the WoS or Scopus.

## 3 RESULTS

### 3.1 *Multilingualism of researchers*

Table 1 displays the numbers and shares of all researchers who published articles throughout the years of 2013 to 2015 in only one language (that is, English, local language, or in another language). As many as 66.5% of Flemish and 60.7% of Norwegian SSH researchers published their articles only in English. 48.3% of Polish and 44.4% of Czech SSH researchers published their articles only in their local language. 3.9% of Czech SSH researchers published their articles only in a language other than English or Czech.

**Table 1:** Researchers (*N* = 51,063) who have published only in English, or their local language(s), or only in another language

Country	Researchers who published only in English		Researchers who published only in local language(s)		Researchers who published only in other languages than English and local language(s)		Total number of researchers
	*n*	%	*n*	%	*n*	%	
Czech Republic	3,105	26.2	5,255	44.4	467	3.9	11,832
Denmark	2,588	57.8	874	19.5	46	1.0	4,479
Finland	1,461	59.3	356	14.4	60	2.4	2,464
Flanders	3,992	66.5	726	12.1	138	2.3	6,006
Norway	1,917	60.7	340	10.8	52	1.6	3,159
Poland	1,659	8.1	9,869	48.3	586	2.9	20,426
Slovenia	731	27.1	734	27.2	74	2.7	2,697
Total	15,453	30.3	18,154	35.6	1,423	2.8	51,063

Similar differences between countries can be observed also in the shares of researchers who published in English, in a local, or in another language at least once. The vast majority of SSH researchers from Denmark, Flanders, Finland, and Norway (79**–**87%) used English as the publication language, whereas less than half of Polish and Czech researchers published in English (44% and 49%, respectively). Only 28.8% of Flemish and 36.0% of Finnish researchers published in their local languages, whereas 88.4% of Polish researchers published in their local language. The highest number of researchers who published in another language was found for Slovenia (14.2%) and the lowest for Denmark (4.2%). Table 2 shows the numbers and shares of researchers who published at least one of their articles in English, in a local, or in another language.

**Table 2:** Researchers (*N* = 51,063) who published in English, local languages, or in another language

Country	Researchers who published in English	Researchers who published in local language(s)			Researchers who published in other language(s)		Total number of researchers
	*n*	%	*n*	%	*n*	%	
Czech Republic	5,772	48.8	8,111	68.6	1,215	10.3	11,832
Denmark	3,532	78.9	1,760	39.3	188	4.2	4,479
Finland	2,037	82.7	887	36.0	151	6.1	2,464
Flanders	5,093	84.8	1,728	28.8	452	7.5	6,006
Norway	2,752	87.1	1,092	34.6	199	6.3	3,159
Poland	9,012	44.1	18,048	88.4	2,261	11.1	20,426
Slovenia	1,797	66.6	1,832	67.9	382	14.2	2,697
Total	29,995	58.7	33,458	65.5	4,848	9.5	51,063

To determine the predictors of multilingualism, we built a binary logistic regression model. This allows us to measure multilingualism in cases where two or more languages have been used by an individual researcher. Our analysis indicates that gender, OECD fields, the number of articles, and the number of journals in which a researcher has published are predictors of multilingualism; however, there can be other factors that we have not measured (see Table S5).

Female researchers (46.6%) use various languages more often than male researchers (45.5%) (OR = 0.88). In addition, researchers from the humanities (44.1%) tend to use fewer languages than researchers from the social sciences (46.9%), but the model shows that the chance of using more languages in the social sciences is smaller (OR = 0.92) than it is in the humanities (which is the reference point in the logistic regression). We also found that researchers who published a greater number of articles (“4–9 articles” and “10 and more articles,” OR = 1.46 and OR = 2.03, respectively) use more languages than researchers who published a smaller number of articles (2–3 articles). Similarly, researchers who published in a greater number of different journals (“3–4 journals” or “5 and more journals,” OR = 2.2 and OR = 3.5, respectively) used more languages than researchers who published in 1–2 journals.

In all seven countries, the majority of SSH researchers publish peer‐reviewed journal articles only in one language, and only a very small share published in three or more languages throughout the 3‐year period (2013–2015). The highest mean number of articles for language variation was found in Slovenia (1.51) and the lowest from Denmark, as well as Flanders 1.22 (see Figure S1). We also found that the highest share of researchers who published in only one language was in Flanders (80.9%), whereas the lowest shares were found in Slovenia (57.2%) and Poland (59.3%). Poland and Slovenia also presented the highest share of researchers publishing in two languages (37.8% and 37.3%, respectively). Moreover, the highest percentage (5.6%) of researchers who published in three or more languages was found in Slovenia.

**Figure 1:**
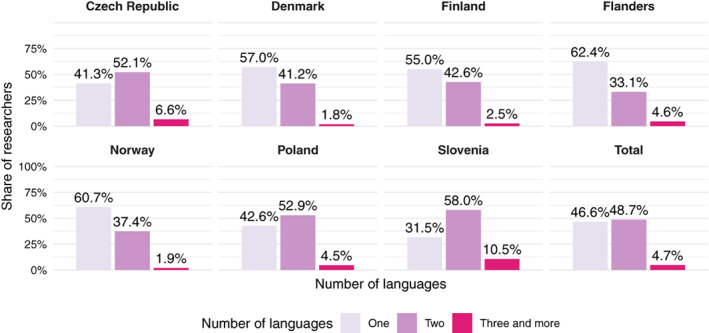
Language patterns of article publishing on the researcher‐level across countries. Researchers who published at least three articles (*N* = 25,365) [Color figure can be viewed at wileyonlinelibrary.com]

This picture is substantially different from what we found in our investigation of the 25,365 researchers (49.7% of the total number of researchers) who published at least three articles throughout 2013 to 2015. Figure 1 presents the shares of SSH researchers in each country who published at least three articles in one, two, and three or more languages throughout the 2013 to 2015 period. Over one‐third of the SSH researchers published in at least two languages in each country. However, in the Czech Republic and Poland this share is over 50% and in Slovenia almost 70%.

**Figure 2:**
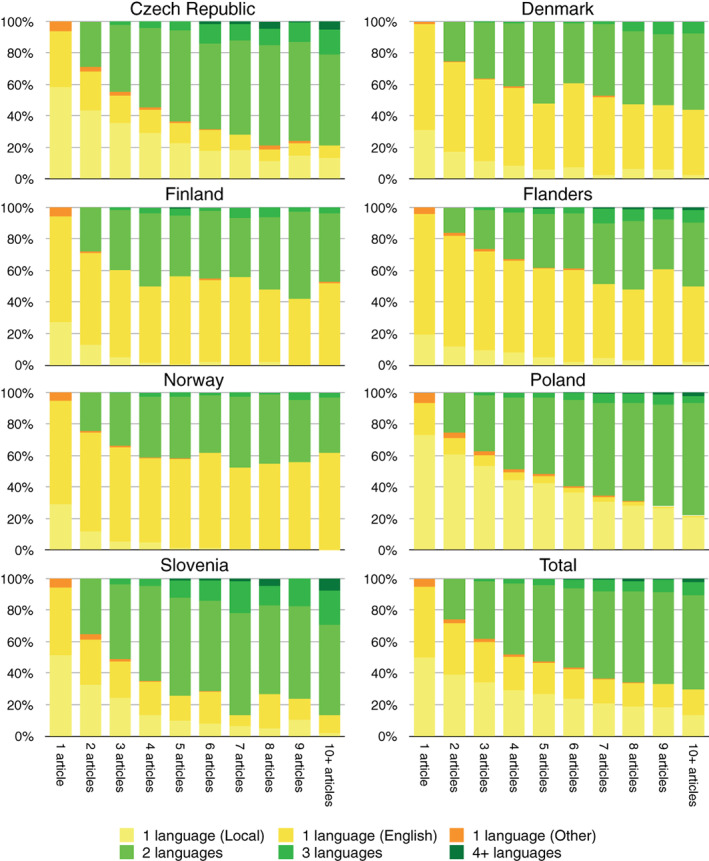
The share of researchers (*N* = 51,063) publishing in 1, 2, 3, or 4 and more languages across groups of researchers with a given number of publications [Color figure can be viewed at wileyonlinelibrary.com]

As expected, researchers who are more productive tend to publish in more languages (cf. Table S5 and Figure S2). However, this picture is much more nuanced. Figure 2 shows that in the Czech Republic, Poland, and Slovenia the share of researchers publishing in at least two languages increases with the corresponding number of articles published by these researchers.

On the other hand, one can see that in Finland, Norway, and to some extent in Denmark and Flanders, the number of publications and the number of languages used are not correlated. Moreover, Figure 2 presents the share of researchers who have used only one language regardless of the number of published publications. We see that the share of researchers who published only in English compared to researchers publishing in local languages is growing as the number of publications increases.

### 3.2 *Country profiles*

The two SSH fields with the largest share of researchers in the seven countries are Economics and Business (18.6%) and Languages and Linguistics (14.0%). There are, however, considerable differences between the countries' SSH profiles (Table S4). While Languages and Linguistics is the second, third, or fourth largest field in all countries (out of 14 OECD field of science categories), Economics and Business is the largest in Poland (27.9%), Flanders (17.8%), Denmark (17.3%), and Slovenia (15.0%) but comes only third in Finland (11.8%), fifth in Norway (9.9%), and sixth in the Czech Republic (8.4%). In the Czech Republic the two largest fields are Political Science (17.9%) and History and Archeology (17.0%), while in Finland, Education (17.2%), and in Norway, Psychology (17.9%) have the largest shares of researchers. Overall, the share of Humanities researchers is somewhat smaller in Denmark, Finland, Flanders, and Norway (29–31%) than in the Czech Republic, Poland, and Slovenia (34–42%).

### 3.3 *Representation of SSH journal publishing*

Earlier we noted that WoS and Scopus provide an impoverished picture of multilingualism in SSH scholarly communication. These data sources, which are often linked to standards of international “excellence,” cover only 25.0% (WoS) and 30.7% (Scopus) of the 164,218 peer‐reviewed journal articles that we focused on in this study. With respect to the English language articles featured in our international sample, the WoS database covered only 58.5%, while Scopus covered only 65.9%. The shortcomings of the two commercial databases can be seen more acutely in the case of our seven European countries' local languages: only 3.4% were covered by WoS and 8.0% covered by Scopus. Moreover, SSH researchers communicate in other non‐English or nonlocal languages, and these too were inadequately covered: WoS (10.6%) and Scopus (17.4%).

### 3.4 *Journal use and publication productivity*

SSH researchers from seven European countries published peer‐reviewed articles in a total of 18,251 journals, of which 5,046 published articles written in local languages. In addition, 3,109 of the journals were used for communicating research results in languages other than English or local languages.

Articles in English were published in 13,164 of the 18,251 journals. However, almost half of those journals were not indexed as source articles in the WoS and Scopus. Each respective database was found to cover 6,736 (51.2%) and 7,695 (58.5%) of the journals that published articles written in English. Moreover, WoS covered only 332 (6.6%) and Scopus only 577 (11.0%) of the journals with local language publications, along with 384 (12.4%) and 551 (17.7%) of the journals that published in other languages.

Local language articles are published mostly in local journals (94.2% of all articles, ranging from 86.0% for Denmark and 98.3% in Poland). We found that in the Czech Republic and Poland the journal/researcher ratio (0.3–0.4) was much smaller than in the other countries (Table 3). Researchers from Norway published in the highest relative number of journals (with a journal/researcher ratio of 1.28), and had the highest mean number of articles (5.3). Nevertheless, productivity does not follow the North/West and Central/East divide, given that the second and third most productive researchers came from Poland and Slovenia, with the lowest mean number of articles observed in the Czech Republic. This result might be influenced by the research evaluation systems in those countries rather than the comprehensiveness of national databases. For instance, when Poland introduced its new publication‐based model, contributions of outstanding value began to diminish. Many articles in low‐tier journals were thus considered more valuable than one article in a very prestigious journal (Kulczycki, 2017).

**Table 3:** The number of researchers, journals, and articles published in the years 2013–2015 across countries

Country	Number of researchers	Number of journals	Journal/Researcher ratio	Number of articles	Articles/Researcher ratio	Mean number of articles per researcher	Maximum number of articles per researcher
Czech Republic	11,832	3,862	0.33	22,514	1.90	2.69	43
Denmark	4,479	3,000	0.67	9,774	2.18	2.77	34
Finland	2,464	2,619	1.06	5,901	2.39	3.32	75
Flanders	6,006	4,666	0.78	13,318	2.22	3.90	71
Norway	3,159	4,050	1.28	11,769	3.73	5.27	160
Poland	20,426	8,153	0.40	92,984	4.55	5.03	96
Slovenia	2,697	2,148	0.80	7,958	2.95	3.98	57
Total	51,063	28,498	0.56	164,218	3.22	4.02	160

### 3.5 *Article languages*

**Figure 3:**
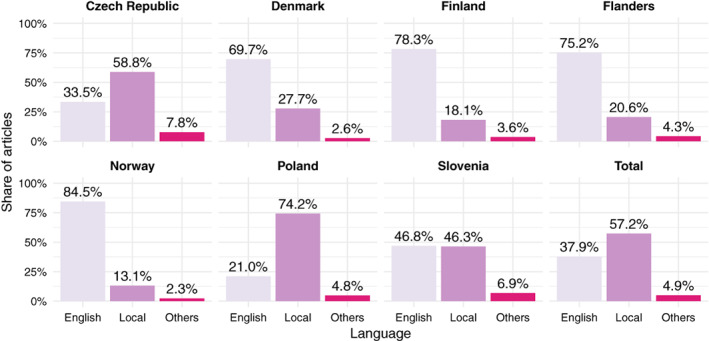
The share of articles per article language across countries [Color figure can be viewed at wileyonlinelibrary.com]

In this part of the study, the focus is on three article language groups: (a) English, (b) local language(s), and (c) other language(s). As expected from our data set, we found that the Nordic and Western European countries—Denmark, Finland, Flanders, and Norway—predominantly used English as a publishing language, while the Central and Eastern European countries, most notably Poland and the Czech Republic, mainly used their local languages (Figure 3). From our sample, both the highest as well as the lowest share of articles published in English were from Norway (84.5% and 13.1% of the total volume, respectively), while the lowest share of English articles and the highest share of local language articles were found in Poland (21.0% and 74.2%, respectively). Slovenia falls in the middle, with almost the same share of English and local language publications. The highest share of publications written in other languages came from the Czech Republic (7.8%) and the lowest were produced in Norway (2.3%).

Northwestern European countries publish mainly in English, but the share of local language publications is much higher in Denmark than in Norway. Figure 5 shows that the share of national language output is the largest in Denmark in almost all fields, but these differences may be explained by the incentives for local language publishing implemented in national research evaluation systems (similar in Denmark and Norway). In Norway, practically all national language channels are placed in the two‐level list on Level 1, while there are at least some at Level 2 in Denmark. Researchers can obtain more points from publications in Level 2 channels than from Level 1.

When we look into the results from Central Europe, we see the share of articles in English is higher in the Czech Republic than in Poland, although the research evaluation regimes and incentives for choosing English publication channels are quite similar in these countries (Good, Vermeulen, Tiefenthaler, & Arnold, 2015; Kulczycki, 2017). One explanation is the size of the Polish scholarly market and the number of users of the Polish language. Researchers from mid‐sized or large countries have a wider audience for publications written in national languages than researchers from countries like the Czech Republic or Slovenia, which have a smaller number of national language users.

While English and local languages were the most frequently used by the seven countries, all of the SSH researchers communicated their research results in a total of 53 different languages. We also found considerable differences between the countries: researchers from Poland published articles in 44 different languages and Flemish researchers published in 13 different languages.

Table 4 indicates the share of most used languages (that is, the top five) for each country, relative to the total number of languages that were used. Sixteen languages have been included in this analysis, and, in addition to English, eight cover the local languages of our seven countries, with seven representing other languages: that is, Croatian, French, German, Italian, Spanish, Slovak, and Russian. For each country, English and German were among the top‐five most‐used languages, with French and Spanish also featured prominently. Russian is among the top‐five languages used in the Czech Republic, Poland, and Finland. None of the local languages were found in the five most‐used languages of another country.

**Table 4:** The five most used languages in each of the seven countries relative to the total number of publications [Color table can be viewed at wileyonlinelibrary.com]

Country	Number of languages	cs	da	de	en	es	fi	fr	hr	it	nl	no	pl	ru	sk	sl	sv
Czech Republic	33	58.8	<0.1	2.3	33.5	0.5	<0.1	0.8	<0.1	0.2	0.1	<0.1	0.7	0.9	1.4	0.1	<0.1
Denmark	16	0	27.7	1.1	69.7	0.5	<0.1	0.3	0	0.1	<0.1	0.2	<0.1	0	0	0	0.2
Finland	25	<0.1	0.1	0.7	78.3	0.3	17.3	0.7	<0.1	0.2	<0.1	0.1	<0.1	0.9	0	0	0.9
Flanders	13	0	0	0.6	75.2	0.7	0	2.5	0	0.2	20.6	0	<0.1	<0.1	0	<0.1	0
Norway	19	0	0.3	0.5	84.5	0.4	<0.1	0.4	<0.1	0.1	<0.1	13.1	<0.1	0.1	0	0	0.3
Poland	44	0.1	0	1.7	21.0	0.3	<0.1	0.6	<0.1	0.3	<0.1	<0.1	74.2	1.0	0.1	<0.1	<0.1
Slovenia	25	0.2	<0.1	1.8	46.8	0.4	0	0.6	1.2	0.6	<0.1	0	0.2	0.4	0.1	46.3	<0.1
Total	53	8.1	1.7	1.5	37.9	0.4	0.6	0.8	0.1	0.3	1.7	1.0	41.1	0.8	0.2	2.3	0.1

*Note*: Colored cells represent the share of the most used publication languages (that is, the top 5) in a given country.

Languages: cs – Czech; da – Danish; de – German; en – English, es – Spanish; fi – Finnish; fr – French; hr – Croatian; it – Italian; nl – Dutch; no – Norwegian; pl – Polish; ru – Russian; sk – Slovak, sl – Slovenian, sv – Swedish.

#### 3.5.1 *Differences between fields*

**Figure 4:**
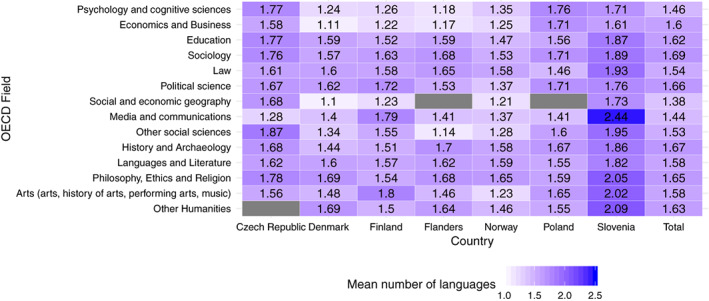
The mean number of languages in which researchers from a given OECD field published articles across countries. Fields are ordered according to the OECD classification. Gray cells indicate that no researcher is assigned to this field in this country. Researchers who published at least three articles (*N* = 25,365) [Color figure can be viewed at wileyonlinelibrary.com]

Language patterns differ across countries as well as across fields. An analysis of the mean number of publication languages shows that in Northern and Western European countries, “Psychology and Cognitive Sciences,” “Economics and Business,” and “Social and Economic Geography” are the most monolinguistic research fields. This lies in stark contrast to Slovenia, and especially Poland, where these fields produce the most multilingual articles. Figure 4 presents the mean number of publishing languages for researchers in a given field based on the classification of the OECD (2007). The results presented in Figure 1 include only those researchers who published at least three articles during the 3‐year period 2013–2015. The highest means were found for Slovenia (in “Media and Communications,” “Other Humanities,” “Philosophy, Ethics, and Religion”) and the lowest ones for Denmark (“Social and Economic Geography” and “Economics and Business”).

**Figure 5:**
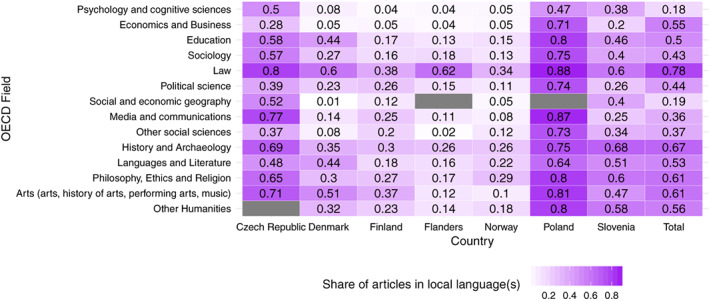
The share of local languages across OECD fields and countries. Fields are ordered according to the OECD classification. Gray cells indicate that no researcher is assigned to this field in this country [Color figure can be viewed at wileyonlinelibrary.com]

Figure 5 illustrates the share of articles in local languages across countries and OECD fields. The highest shares occur in the fields of “Law,” “History and Archeology,” and “Arts.” The lowest shares were observed for “Psychology and Cognitive Sciences” and “Economics and Business” (except for Poland).

**Figure 6:**
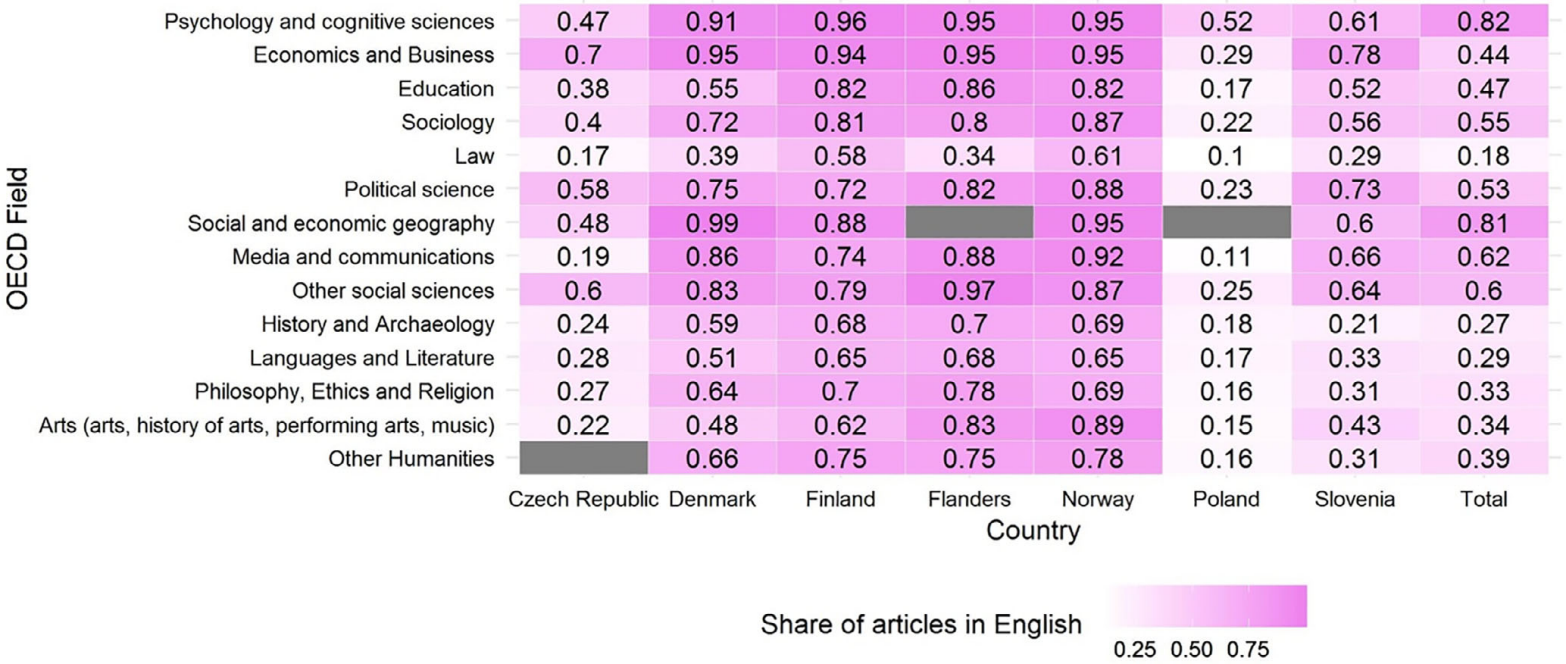
The share of articles in English across OECD fields and countries. Fields are ordered according to the OECD classification. Gray cells indicate that no researcher is assigned to this field in this country [Color figure can be viewed at wileyonlinelibrary.com]

Figure 6 displays the share of articles in English across countries and fields. The highest shares occur in the fields of “Economics and Business,” “Psychology and Cognitive Sciences,” and “Social and Economic Geography” in all countries. The lowest share is for “Law.” The three gray blocks indicate cases where a particular field is not assigned to any researcher from the country.

**Figure 7:**
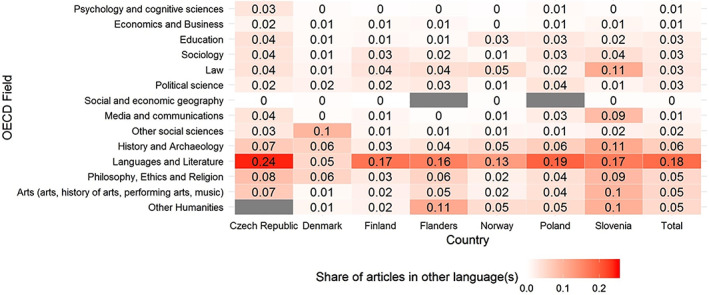
The share of articles in other language(s) across OECD fields and countries. Fields are ordered according to the OECD classification. Gray cells indicate that no researcher is assigned to this field in this country [Color figure can be viewed at wileyonlinelibrary.com]

Figure 7 displays the share of articles in other languages across OECD fields and countries. The highest shares in all countries (with the exception of Denmark) relate to “Languages and Literature.”

#### 3.5.2 *Languages and collaboration patterns*

Finally, we indicate how single‐ or multiauthored articles are related to publication language (Table 5). The ratio of multi/single‐authored articles is highest for Norway and Flanders, where multiauthored articles are more than twice as frequent as single‐authored articles. The lowest ratio is for Poland (0.30), where coauthorship is not as common as in other countries. The ratio of multi/single‐authored articles according to article languages shows that for all countries (except Poland) articles in English were more often written by multiple authors rather than one author. We also found that, for all countries, articles that were written in local and other languages were more often single‐authored than multiauthored.

**Table 5:** Single‐authored and multiauthored articles written either in English, a local language, or another language across countries

Country	Number of articles			Multi/single‐authored article ratio according to article language		
	Single‐authored	Multiauthored	Multi/single‐authored article ratio	English	Local(s)	Other(s)
Czech Republic	14,465	8,049	0.56	1.16	0.38	0.24
Denmark	5,223	4,551	0.87	1.25	0.35	0.36
Finland	2,303	3,598	1.56	2.09	0.63	0.43
Flanders	4,244	9,074	2.14	3.49	0.65	0.62
Norway	3,455	8,314	2.41	3.37	0.57	0.40
Poland	71,645	21,339	0.30	0.66	0.23	0.17
Slovenia	4,411	3,547	0.80	1.81	0.40	0.21
Total	105,746	58,472	0.55	1.43	0.28	0.23

## 4 DISCUSSION

Our study shows that multilingual publishing is an ongoing practice in many SSH research fields regardless of geographical location, political situation, and/or historical heritage. Even in countries like Denmark, Finland, Flanders, and Norway, where the majority of articles are published in English, a substantial share of researchers have published in two or more languages. In countries like the Czech Republic, Poland, and Slovenia, where it is more common to use the local language in publishing, a considerable share of SSH researchers publish also in English. Nevertheless, for all countries, a greater balance with respect to publication languages is possible and desirable. If a larger share of SSH scholars published in both their local languages and in English, there is more potential to link this work to societal problems, at least to local societies, but at the same time, the international community definitely benefits when a larger share of scholars engage in English‐language communication.

This research calls upon international and national research policy and evaluation regimes to more fully recognize the value of multilingualism in scholarly communication, in order to foster a balance between the demands of international excellence and local relevance (Kulczycki, Mustajoki, Pölönen, & Røeggen, 2019; Robinson‐Garcia & Ràfols, in press; Sivertsen, 2018). A great deal of original research coming from the SSH may be applied to local issues, pertaining to the local language, heritage, and culture. Moreover, certain forms of research (for example, research in art history) can be of international relevance and can have international impact when published in languages other than English. However, increasing our focus on local language publishing requires more than just the simple application of metrics. All seven countries included in this study have already moved beyond the use of the WoS and Scopus, by relying (also) on comprehensive institutional publication data to support evaluations within their performance‐based funding systems. We can still recognize the value of local language publishing in all metric and expert‐based evaluation approaches, but this needs to include cooperation from research organizations as well, where internal funding models, hiring, promotion, and funding decisions are made, as well as international and national project funding.

For the most part, it has been the researchers from our seven countries that have taken responsibility for communicating locally relevant research in their local languages. Czech, Danish, Finnish, Norwegian, Polish, and Slovene are rather marginal publication languages, and not likely to be put to use by researchers in any other country (unless there is some cross‐country coauthorship). Even Dutch and Swedish, which are primarily spoken within the Netherlands and Sweden, constitute small languages in terms of number of speakers. As such, the importance attached to promoting local language publishing is arguably greater for the countries that we focus on in this study than it is for countries like France, Germany, or Spain, where there are greater possibilities for the local language to have an international reach (for example, publications written in Spanish are attractive to researchers working in South America).

One way of making research results published in English more readily accessible to citizens is to publish the same results in a local language, but in a more popularized format, for instance, via a blog or alternative news source. This practice could, however, be at odds with current regulations concerning self‐plagiarism. For example, “Re‐publishing substantive parts of one's own earlier publications, including translations, without duly acknowledging or citing the original” (All European Academies [ALLEA], 2011), is considered to be an “unacceptable practice” under the European Code of Conduct for Research Integrity. It has been pointed out that some evaluation regimes also have rules against double counting in evaluation procedures (Dahler‐Larsen, 2017). An international discussion is needed to determine more clearly how this type of publication strategy could be seen as beneficial, rather than a violation of research integrity and publication counting (Israel, 2019).

Sustaining a balanced approach to multilingual scholarly communication also requires a healthy infrastructure for local language publishing. The social and cultural context of local language journals is unique: they cannot be replaced by publication channels produced in other countries, or by international mega‐journals. In addition to communicating research results to local audiences, national journals also maintain local research communities. In some smaller countries, the market for local language publishing could be too small for commercial publishers to get involved. Therefore, national journals are often not‐for‐profit and published by research institutions or learned societies. They may not be able to transition to an open access publishing model without losing income from subscriptions and membership fees (Wise & Estelle, 2019). One way to enable open access publishing for national, local‐language journals is to create a specific platform for hosting and maintaining the most important local journals, an example of which has been recently implemented in Norway (Sivertsen, 2018).

## 5 CONCLUSION

Research is international, but multilingual publishing keeps locally relevant research alive with the added potential for creating impact. As a result of our seven‐country European study, we have found that multilingual scholarly communication is demonstrably alive and well across SSH, despite the fact that we argue for a greater balance between English and local language publishing. Both are not only possible but desirable. International and national research policy and evaluation regimes need to foster a greater balance between the demands of international excellence and the local relevance of research. In stating this, we also wish to highlight the recommendations of the Helsinki Initiative on Multilingualism in Scholarly Communication. The aim of this initiative is to support the dissemination of research results for the full benefit of the society, to protect national infrastructures for publishing locally relevant research, and to promote language diversity in research assessment, evaluation, and funding systems.

## AUTHOR CONTRIBUTIONS

E.K., R.G., J.P., and T.C.E.E. designed the study. E.K. and E.R analyzed the data and constructed the figures. E.K., R.G., J.P., T.C.E.E., and A.A.Z. wrote the article. All authors created the data sets, read, and revised the article.

## DATA AVAILABILITY

The data on journals and researchers from the national databases were obtained thanks to the agreements across authors and the database owners and in the raw form cannot be shared. Nonetheless, the data associated with this study aggregated on the anonymized field‐level are available from the corresponding author upon request.

## Supporting information


**Appendix S1.** Supporting Information.
