# Supplementary material for: Multilingual publishing in the social sciences and humanities: A seven‐country European study
Source: J Assoc Inf Sci Technol. 2020 Jan 22;71(11):1371–85. doi: 10.1002/asi.24336 (PMC7687152; doi:10.1002/asi.24336)
Supplement: Supplementary file 1 — Appendix S1. Supporting Information. [file ASI-71-1371-s001.docx]

Supplementary information for:
**Multilingual publishing in the social sciences and humanities: A seven-country European study**

Emanuel Kulczycki, Raf Guns, Janne Pölönen, Tim C.E. Engels, Ewa A. Rozkosz, Alesia A. Zuccala, Kasper Bruun, Olli Eskola, Andreja Istenič Starčič, Michal Petr, Gunnar Sivertsen

**Supplementary Methods**

**Researchers**

Supplementary Table 1 provides information on a typology of researches included in the study. Below, we list the set of researchers considered for each country in the study.

- Czech Republic: Researchers from all Czech universities, basic and applied sciences institutes.
- Denmark: Researchers from all eight Danish universities.
- Flanders: Researchers from all five Flemish universities, who are affiliated to a unit (research group, department, or faculty) classified as SSH. Emeriti and PhD researchers are included insofar as their publications mention an affiliation to a Flemish university.
- Finland: Researchers from 5 out of 14 universities, of whose FTE in the years 2011–2012 at least 50 % was allocated to SSH fields according to the personnel and publication information provided to the Ministry of Education and Culture.
- Norway: Researchers from the 4 largest (out of a total of 10) Norwegian universities who published at least three times (but not only articles) in the years 2011–2017.
- Poland: Researchers employed by all Polish universities, basic and applied sciences institutes. In the study, we include all PhD level researchers who attributed SSH fields (mostly on the basis of their PhD) in the Polish current research information system.
- Slovenia: Researchers from all Slovenian universities, basic and applied sciences institutes and other researchers who are registered in SICRIS system.

Supplementary Table 1. Typology of researchers included in the study.

| Database / country | Researchers included in the data | | | |
| --- | --- | --- | --- | --- |
|  | Full-time | Part-time | Emeritus | PhD researchers |
| Czech Republic – The National Registry of RD & I Outputs | + | + | + | + |
| Denmark – The Danish Bibliometric Research Indicator (Denmark) | + | + | + | + |
| Finland – The VIRTA Publication Information Service | + | + | – | + |
| Flanders – The Flemish Academic Bibliographic Database for the Social Sciences and Humanities | + | + | + | + |
| Norway – The Norwegian Science Index | + | + | + | + |
| Poland – The Polish Scholarly Bibliography | + | + | + | – |
| Slovenia – The Slovenian Current Research Information System | + | + | + | – |

**Field Classification**

Using the Daraio and Glänzel (2016) categorization, subject classifications can be divided into four categories: (1) *cognitive* – content–related, (2) *administrative* – responsibility-related, (3) *organizational –* structure-related, and (4) *qualification-based* – competency-related. Supplementary Table 1 shows the type of subject classification systems used in each of the databases.

Supplementary Table 2. Field classification systems used in each of the databases for assigning researchers to fields

| Database / country | Classification system | | | |
| --- | --- | --- | --- | --- |
|  | Cognitive | Administrative | Organizational | Qualification-based |
| Czech Republic – The National Registry of RD & I Outputs | + | – | – | – |
| Denmark – The Danish Bibliometric Research Indicator (Denmark) | + | – | – | – |
| Finland – The VIRTA Publication Information Service | – | – | – | + |
| Flanders – The Flemish Academic Bibliographic Database for the Social Sciences and Humanities | – | – | + | – |
| Norway – The Norwegian Science Index | + | – | – | – |
| Poland – The Polish Scholarly Bibliography | – | – | – | + |
| Slovenia – The Slovenian Current Research Information System | + | – | – | – |

Variants of a cognitive classification have been used for four countries, i.e. the Czech Republic, Denmark, Norway, and Slovenia. It means that fields are assigned to researchers on the basis of the content of articles or journals in which those researchers published. For instance, in Slovenia researchers were assigned to fields on the basis of their article portfolio (e.g. if the majority of articles authored by a given researcher are classified as papers in Economics and Business, then this researcher is classified to this field). In Finland and Poland, qualification-based classifications were used: Finnish researchers were classified as SSH researchers when at least half of their work-time was dedicated for SSH fields at a university. In Flanders, publications are assigned according to the organizational structure of the universities to which the authors of the publications are affiliated. A qualification-based classification was used for the data from Finland and Poland. No researchers are assigned to ‘Other humanities’ in the Czech Republic and no researchers to ‘Social and economic geography’ in Flanders and Poland.

## Peer-Reviewed Articles

The data includes peer-reviewed journal articles from the publication years 2013–2015, except for Finnish articles, which are—because of the data availability—from the period 2012–2014.

Supplementary Table 3 shows which method was used to identify peer-reviewed articles for each country.

Supplementary Table 3. Methods of identifying peer-reviewed articles

| Country / Database | Method of identifying peer-reviewed articles | | | |
| --- | --- | --- | --- | --- |
|  | Authors’ self-report | | Authority list | |
| Czech Republic – The National Registry of RD & I Outputs | | + | | – |
| Denmark – The Danish Bibliometric Research Indicator (Denmark) | | – | | + |
| Finland – The VIRTA Publication Information Service | | + | | – |
| Flanders – The Flemish Academic Bibliographic Database for the Social Sciences and Humanities | | – | | + |
| Norway – The Norwegian Science Index | | – | | + |
| Poland – The Polish Scholarly Bibliography | | + | | – |
| Slovenia – The Cooperative online bibliographic system and services | | – | | + |

**Datasets**

For the purposes of this study, we analyze the set of all peer-reviewed SSH articles and the set of SSH researchers broken down into OECD fields and countries. We use five datasets extracted from the full set to (A) present the total number of researchers and their productivity in terms of the number of articles across fields and countries, (B) compare the shares of article languages across seven countries, (C) analyze language patterns at the article level, (D) describe language patterns of journal article publishing across fields and countries from the researcher-level perspective, and (E) present the differences in the use of language between single-authored and multi-authored articles. The five datasets we used are described below:

1. Dataset A contains statistics for 164,218 articles from the years 2013–2015 registered in at least one of the seven databases. For our analysis of the total number of SSH researchers and their productivity, we used the number of articles per country and the number of scholarly peer-reviewed journals in which researchers published as our units of analysis.
2. Dataset B contains statistics for 164,218 articles from the years 2013–2015 with a nominal variable related to article languages: English, local language(s), other languages(s).
3. Dataset C contains statistics for 164,218 articles aggregated at the OECD fields level. Because 41,890 articles have been authored by two or more researchers from different OECD fields, there are in total 206,108 article–field combinations. For our analysis of language patterns at the article-level, we used the share of articles in each of countries published in three groups of languages: English, local language(s), other language(s).
4. Dataset D contains statistics for 51,063 researchers who published at least one article in the years 2013–2015 and their 206,108 article–field combinations aggregated at the OECD fields level. 811 researchers who published 3,967 articles have no specific OECD field assigned although they were classified as SSH researchers. For analysis of language patterns at the researcher level, we calculated in how many languages a given researcher published their articles, distinguishing between one, two, and three and more. For the analysis of the mean number of languages used across countries and OECD fields, we calculated the means of the number of languages used by researchers in a given field or country.
5. Dataset E contains statistics for 164,218 articles from the years 2013–2015 with a rank variable related to each article (single-authored, multi-authored) and a nominal variable related to its language (English, local language, other language).

**Journals**

Journals in the data are uniquely identified by ISSN or eISSN. On the basis of the ISSNs, we have assigned to each journal the ISSN-L retrieved from the ISSN International Register. The ISSN-L helps us to identify all unique journals used by SSH researchers included in the study and to analyze the journal data at the eight-country level.

Using the ISSN-L, we have added information whether a given journal is indexed in the Web of Science (i.e., the Science Citation Index Expanded, the Social Science Citation Index, the Arts & Humanities Citation Index, and the Emerging Sources Citation Index), and Scopus.

**Supplementary Results**

Supplementary Figure 1 shows the number of languages in which researchers publish their articles across countries.


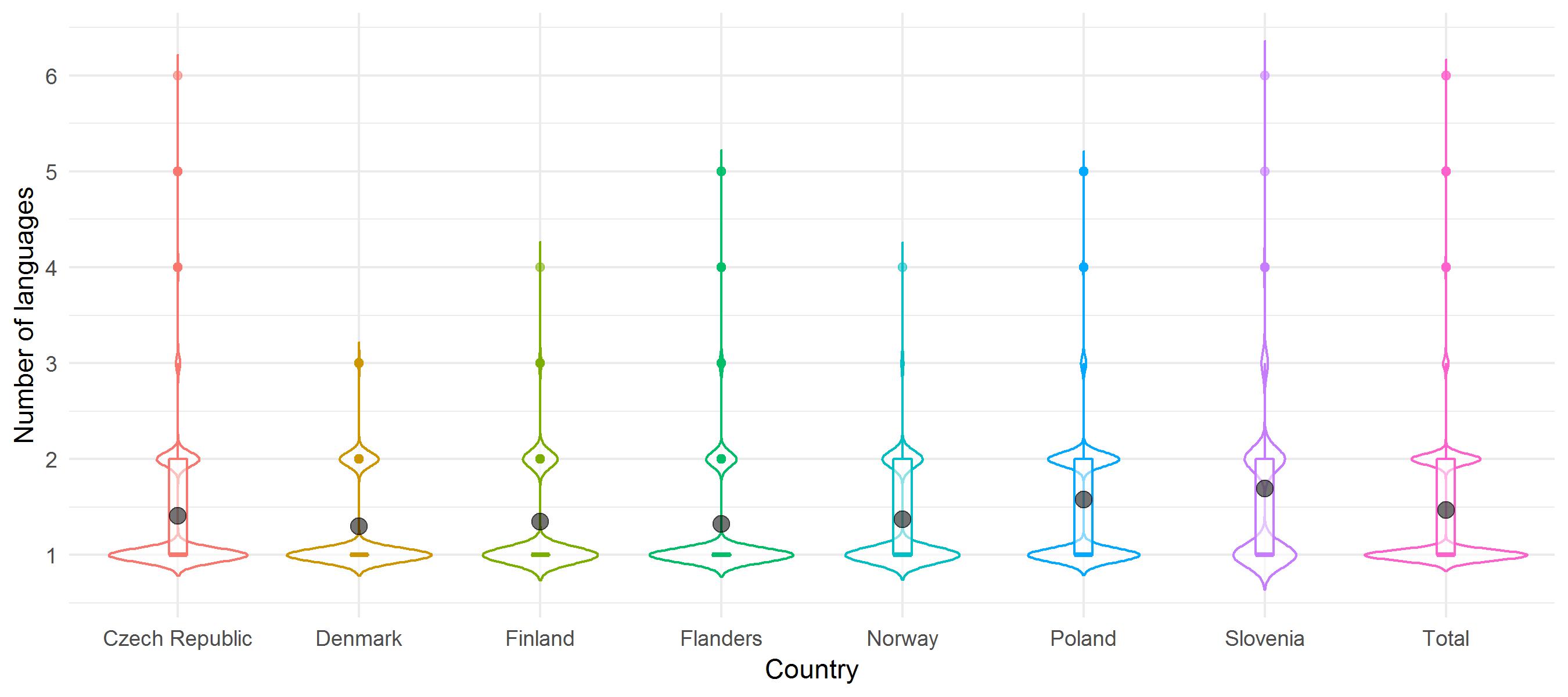


*Supplementary Figure 1.* Violin plot of the number of languages in which researchers published their articles across countries. The black dots represent the mean.

Supplementary Figure 2 shows that, on average, researchers published 4.02 articles, ranging from an average of 5.03 for Norway to 2.69 for the Czech Republic. Comparing these mean values with an article-researcher ratio, one can find that the highest value for this ratio is for Poland (4.55) whereas the lowest occurs for Norway (3.73). Hence, the higher mean number of articles per researcher in Norway results from more multi-authored publications.


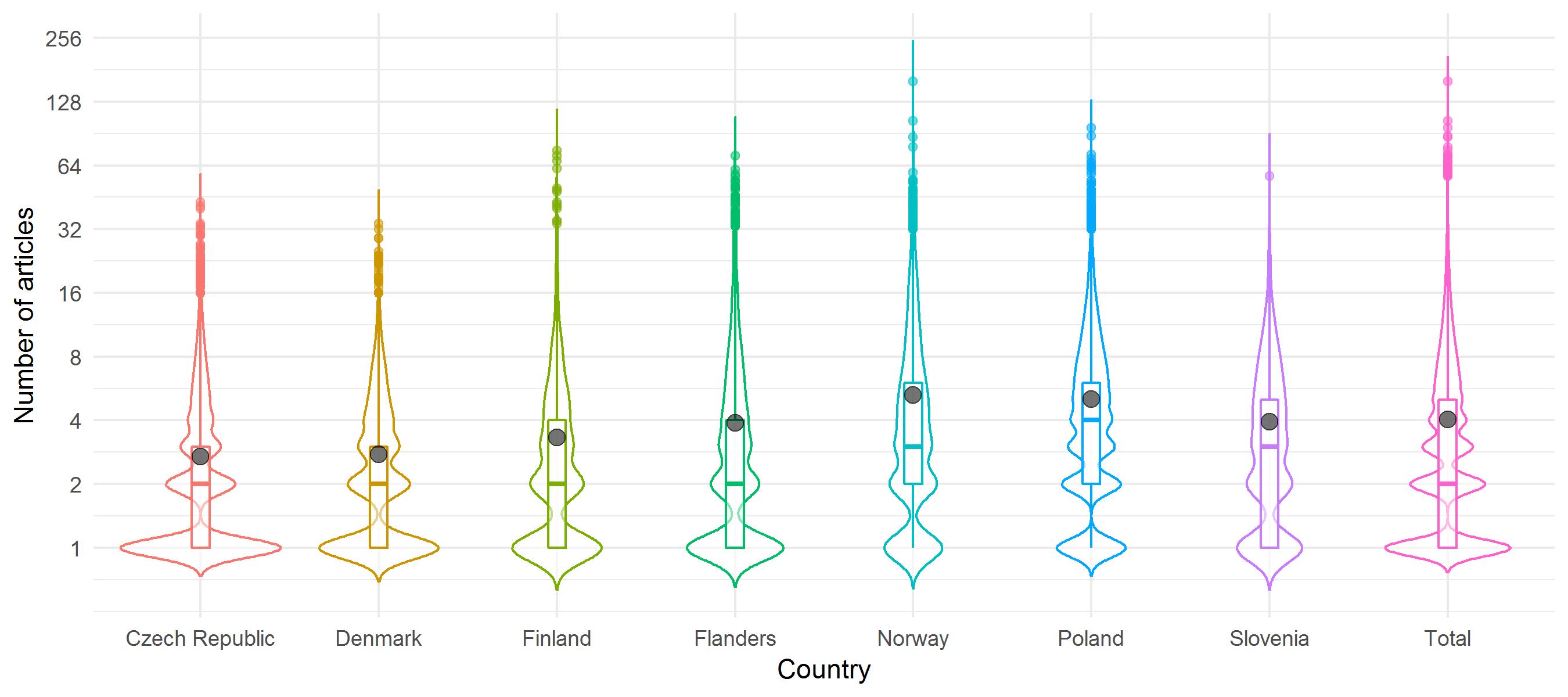


*Supplementary Figure 2.* Violin plot of the number of articles published by researchers across countries. Black dots represent the mean values per country.

Supplementary Table 4 presents the number of researchers who published at least one article in the years 2013–2015 across OECD fields and countries.

Supplementary Table 4. The number and share of researchers who published at least one article in the years 2013–2015 across OECD fields and countries

|  | Czech Republic | | Denmark | | Finland | | Flanders | | Norway | | Poland | | Slovenia | | All countries | | |
| --- | --- | --- | --- | --- | --- | --- | --- | --- | --- | --- | --- | --- | --- | --- | --- | --- | --- |
| OECD Field | *n* | % | *n* | % | *n* | % | *n* | % | *n* | % | *n* | % | *n* | % | | *n* | % |
| Arts (arts, history of arts, performing arts, music) | 1,000 | 8.5 | 146 | 3.3% | 17 | 0.7 | 250 | 4.2 | 93 | 2.9 | 391 | 2.0 | 111 | 4.3 | | 2,008 | 4.0 |
| Economics and Business | 999 | 8.4 | 776 | 17.3 | 290 | 11.8 | 1,072 | 17.8 | 314 | 9.9 | 5,509 | 27.9 | 383 | 15.0 | | 9,343 | 18.6 |
| Education | 1,500 | 12.7 | 376 | 8.4 | 425 | 17.2 | 381 | 6.3 | 337 | 10.7 | 1,289 | 6.5 | 276 | 10.8 | | 4,584 | 9.1 |
| History and Archaeology | 2,010 | 17.0 | 228 | 5.1 | 144 | 5.8 | 303 | 5.0 | 188 | 6.0 | 1,619 | 8.2 | 224 | 8.8 | | 4,716 | 9.4 |
| Languages and Literature | 1,240 | 10.5 | 547 | 12.2 | 313 | 12.7 | 646 | 10.8 | 375 | 11.9 | 3,586 | 18.2 | 331 | 13.0 | | 7,038 | 14.0 |
| Law | 774 | 6.5 | 280 | 6.3 | 120 | 4.9 | 776 | 12.9 | 187 | 5.9 | 2,082 | 10.5 | 146 | 5.7 | | 4,365 | 8.7 |
| Media and communications | 120 | 1.0 | 263 | 5.9 | 68 | 2.8 | 238 | 4.0 | 120 | 3.8 | 265 | 1.3 | 35 | 1.4 | | 1,109 | 2.2 |
| Other Humanities | 0 | 0.0 | 214 | 4.8 | 111 | 4.5 | 104 | 1.7 | 76 | 2.4 | 352 | 1.8 | 75 | 2.9 | | 932 | 1.9 |
| Other social sciences | 347 | 2.9 | 226 | 5.0 | 187 | 7.6 | 356 | 5.9 | 127 | 4.0 | 550 | 2.8 | 44 | 1.7 | | 1,837 | 3.7 |
| Philosophy, Ethics and Religion | 676 | 5.7 | 246 | 5.5 | 184 | 7.5 | 414 | 6.9 | 179 | 5.7 | 1,347 | 6.8 | 126 | 4.9 | | 3,172 | 6.3 |
| Political science | 2,119 | 17.9 | 369 | 8.2 | 113 | 4.6 | 259 | 4.3 | 152 | 4.8 | 928 | 4.7 | 250 | 9.8 | | 4,190 | 8.3 |
| Psychology and cognitive sciences | 531 | 4.5 | 288 | 6.4 | 171 | 6.9 | 889 | 14.8 | 564 | 17.9 | 872 | 4.4 | 108 | 4.2 | | 3,423 | 6.8 |
| Social and economic geography | 119 | 1.0 | 100 | 2.2 | 54 | 2.2 | 0 | 0.0 | 118 | 3.7 | 0 | 0.0 | 86 | 3.4 | | 477 | 0.9 |
| Sociology | 397 | 3.4 | 420 | 9.4 | 267 | 10.8 | 318 | 5.3 | 329 | 10.4 | 967 | 4.9 | 360 | 14.1 | | 3,058 | 6.1 |
| Total | 11,832 | 100.0 | 4,479 | 100.0 | 2,464 | 100.0 | 6,006 | 100.0 | 3,159 | 100.0 | 19,757 | 100.0% | 2,555 | 100.0 | | 50,252 | 100.0 |

Supplementary Table 5 presents the results of the logistic regression of researchers who are and who are not multilinguals across gender, OECD fields, the number of articles, and the number of journals in which those researchers published.

Supplementary Table 5. The logistic regression of researchers who are and who are not multilinguals across gender, OECD fields, the number of articles, and the number of journals in which those researchers published

| Parameter | *n* | No multilingualism | | Multilingualism | | Odds ratio | 95% confidence interval for odds ratio | *p*-value |
| --- | --- | --- | --- | --- | --- | --- | --- | --- |
|  |  | *n* | % | *n* | % |  |  |  |
| Gender | | | | | | | | |
| Female | 15677 | 8,375 | 53.4 | 7,302 | 46,6 | 1 | (Ref.) | < .001 |
| Male | 18,594 | 10,149 | 54.6 | 8,445 | 45.4 | .88 | (.84; .92) | < .001 |
| OECD field | | | | | | | | |
| Humanities | 11,584 | 6,478 | 55.9 | 5,106 | 44.1 | 1 | (Ref.) | < .001 |
| Social Sciences | 22,687 | 12,046 | 53.1 | 10,641 | 46.9 | .92 | (.88; .97) | < .001 |
| Number of articles | | | | | | | | |
| 2–3 articles | 15,773 | 10,894 | 69.1 | 4,879 | 30.1 | 1 | (Ref.) | < .001 |
| 4–9 articles | 14,324 | 6,382 | 44.6 | 7,942 | 55.4 | 1.46 | (1.37; 1.56) | < .001 |
| 10 and more articles | 4,174 | 1,248 | 29.9 | 2,926 | 70.1 | 2.03 | (1.84; 2.25) | < .001 |
| Number of journals | | | | | | | | |
| 1–2 journals | 13,666 | 9,890 | 72.4 | 3,776 | 27.6 | 1 | (Ref.) | < .001 |
| 3–4 journals | 11,467 | 5,656 | 49.3 | 5,811 | 50.7 | 2.2 | (2.07; 2.34) | < .001 |
| 5 and more journals | 9,138 | 2,978 | 32.6 | 6,160 | 67.4 | 3.5 | (3.22; 3.81) | < .001 |

*Note.* R^2^ = .084 (Hosmer-Lemeshow), .11 (Cox-Snell), .147 (Negelkerke). Model χ^2^(6) = 3985.666, *p* < .001.

**References**

Daraio, C. & Glänzel, W. (2016). Grand challenges in data integration—state of the art and future perspectives: An introduction. *Scientometrics,* *108*(1)**,** 391–400. https://doi.org/10.1007/s11192-016-1914-5

Organisation for Economic Co-Operation and Development. (2007). *Revised field of science and technology (FOS) classification in the frascati manual DSTI/EAS/STP/NESTI(2006)19/FINAL*. Retrieved from http://www.oecd.org/science/inno/38235147.pdf
